# *Plasmodium falciparum* Hop (PfHop) Interacts with the Hsp70 Chaperone in a Nucleotide-Dependent Fashion and Exhibits Ligand Selectivity

**DOI:** 10.1371/journal.pone.0135326

**Published:** 2015-08-12

**Authors:** Tawanda Zininga, Stanely Makumire, Grace Wairimu Gitau, James M. Njunge, Ofentse Jacob Pooe, Hanna Klimek, Robina Scheurr, Hartmann Raifer, Earl Prinsloo, Jude M. Przyborski, Heinrich Hoppe, Addmore Shonhai

**Affiliations:** 1 Department of Biochemistry, School of Mathematics and Natural Sciences, University of Venda, Thohoyandou, 0950, South Africa; 2 Department of Biochemistry & Microbiology, University of Zululand, P. Bag X1001, KwaDlangezwa, 3886, South Africa; 3 Department of Biochemistry and Microbiology, Rhodes, Grahamstown, 6140, South Africa; 4 Flow cytometry core facility, Institute for Medical Microbiology, University Clinic Marburg, Marburg, Germany; 5 Parasitology, FB Biology, Philipps University Marburg, 35043, Marburg, Germany; 6 Biotechnology Innovation Centre, Rhodes University, Grahamstown, 6140, South Africa; Institut national de la santé et de la recherche médicale—Institut Cochin, FRANCE

## Abstract

Heat shock proteins (Hsps) play an important role in the development and pathogenicity of malaria parasites. One of the most prominent functions of Hsps is to facilitate the folding of other proteins. Hsps are thought to play a crucial role when malaria parasites invade their host cells and during their subsequent development in hepatocytes and red blood cells. It is thought that Hsps maintain proteostasis under the unfavourable conditions that malaria parasites encounter in the host environment. Although heat shock protein 70 (Hsp70) is capable of independent folding of some proteins, its functional cooperation with heat shock protein 90 (Hsp90) facilitates folding of some proteins such as kinases and steroid hormone receptors into their fully functional forms. The cooperation of Hsp70 and Hsp90 occurs through an adaptor protein called Hsp70-Hsp90 organising protein (Hop). We previously characterised the Hop protein from *Plasmodium falciparum* (PfHop). We observed that the protein co-localised with the cytosol-localised chaperones, PfHsp70-1 and PfHsp90 at the blood stages of the malaria parasite. In the current study, we demonstrated that PfHop is a stress-inducible protein. We further explored the direct interaction between PfHop and PfHsp70-1 using far Western and surface plasmon resonance (SPR) analyses. The interaction of the two proteins was further validated by co-immunoprecipitation studies. We observed that PfHop and PfHsp70-1 associate in the absence and presence of either ATP or ADP. However, ADP appears to promote the association of the two proteins better than ATP. In addition, we investigated the specific interaction between PfHop TPR subdomains and PfHsp70-1/ PfHsp90, using a split-GFP approach. This method allowed us to observe that TPR1 and TPR2B subdomains of PfHop bind preferentially to the C-terminus of PfHsp70-1 compared to PfHsp90. Conversely, the TPR2A motif preferentially interacted with the C-terminus of PfHsp90. Finally, we observed that recombinant PfHop occasionally eluted as a protein species of twice its predicted size, suggesting that it may occur as a dimer. We conducted SPR analysis which suggested that PfHop is capable of self-association in presence or absence of ATP/ADP. Overall, our findings suggest that PfHop is a stress-inducible protein that directly associates with PfHsp70-1 and PfHsp90. In addition, the protein is capable of self-association. The findings suggest that PfHop serves as a module that brings these two prominent chaperones (PfHsp70-1 and PfHsp90) into a functional complex. Since PfHsp70-1 and PfHsp90 are essential for parasite growth, findings from this study are important towards the development of possible antimalarial inhibitors targeting the cooperation of these two chaperones.

## Introduction

Malaria remains a major killer disease especially in sub-Saharan Africa. The disease is caused by obligate protozoan parasites of the genus *Plasmodium*. Malaria parasites are transferred into humans during a blood meal by mosquito vectors. Once in the host they travel to the liver cells and eventually infect red blood cells. It is at the blood stage that malaria parasites manifest malaria pathology by modifying the infected erythrocytes making them cytoadherent [1]. It is intriguing how malaria parasites survive under variable physiological conditions prevailing in the warm blooded host following their introduction from cold blooded mosquito vectors. Of particular interest is how the parasite maintains the structural integrity of its proteome under the varied and harsh environmental conditions it encounters in the host. It has been suggested that heat shock proteins (Hsps) may assist the parasite survive the challenges that it faces in the host through their role in the maintenance of proteostasis [2,3]. Heat shock proteins fall under the molecular chaperone class of molecules as they oversee the folding of other proteins in the cell. Consequently, the expression of certain parasite Hsps is induced in response to stress [4,5] in order to manage the additional burden of facilitating protein folding under unfavourable conditions. Heat shock protein 70 (Hsp70) is one of the most well studied Hsps and its role in the survival and pathogenicity of malaria parasites has been documented [3,6]. Although Hsp70 is known to facilitate folding of certain proteins into their fully functional forms, it partners with Hsp90 to fold some proteins such as kinases and steroid hormone receptors [7].

*Plasmodium falciparum* Hsp70 (PfHsp70-1) and PfHsp90 are essential chaperones that are located in the parasite cytosol [5,8]. Both proteins are prospective antimalarial targets [9] as their inhibition by small molecules leads to parasite death [8,10]. Thus, some Hsps of plasmodial origin, amongst them Hsp90 and Hsp70 are deemed potential antimalarial candidates. Plasmodial Hsps also appear to be attractive antimalarial targets especially in combination therapies. For example, a study that was conducted using a mouse malaria model, showed that an inhibitor of parasite Hsp90 complemented the antimalarial action of chloroquine [11]. In addition, the expression of both PfHsp70 and PfHsp90 by malarial parasites at the clinical phase of the disease has been reported to correlate with disease prognosis [12], suggesting that they play an important role in pathogenicity. Given the role of these two chaperones in the development of the malaria parasite, it is important to establish how their functions are coordinated.

In other systems, the function of Hsp70 and Hsp90 (S1 Fig) is coordinated by a protein known as Hsp70-Hsp90 organising protein (Hop), which is also known as Sti1 [13]. Hop serves as an adaptor protein that brings Hsp70 and Hsp90 in a functional complex. Hop contains three tetratricopeptide repeats (TPR): TPR1, TPR2A and TPR2B (S1 Fig) [14]. The C-terminal EEVD motif of Hsp70 and Hsp90 is crucial for its interaction with Hop [15,16,17]. The TPR1 domain of Hop binds to Hsp70, and on the other hand, Hop interacts with Hsp90 via the TPR2A subdomain [16]. The role of the TPR2B subdomain of Hop remains largely unresolved. However, it has been suggested that this motif could also bind Hsp70 [16]. It has been proposed that Hop interacts with Hsp70 and Hsp90 either through the TPR1-TPR2A or TPR2B-TPR2A modules, respectively [16]. Hop is thought to bind two molecules of Hsp70 in the absence of Hsp90, and only one Hsp70 molecule is bound to Hop when Hsp90 is present [17]. However, it has recently been proposed that the TPR2B subdomain of Hop exhibits high affinity for Hsp70 in the absence of Hsp90 [18]. Based on the latter hypothesis, it is thought that binding of Hsp90 shifts Hsp70’s preference from binding TPR2B to TPR1. Consequently, it is believed that Hop binds to Hsp70 in a 1:1 molar ratio [18]. In light of the prevailing contrasting views on the mechanism of action of Hop it is crucial to fully elucidate the structure-function features of Hop that facilitates its interaction of Hsp70 and Hsp90.

In a previous study, we described a *P*. *falciparum* Hsp70-Hsp90 organising protein (PfHop) which co-localises with both PfHsp70-1 and PfHsp90 in the parasite cytosol [19]. Based on size exclusion chromatographic analysis, we previously reported the existence of PfHop together with PfHsp90 and PfHsp70-1 in a complex [19]. However, as yet there is no evidence for the direct interaction of PfHop and PfHsp70-1. In addition, the roles of the TPR motifs of PfHop in mediating its interaction with both PfHsp70-1 and PfHsp90 remain unresolved. The findings from the current study suggest that PfHop, like PfHsp70-1 is heat inducible and that the two directly physically associate. Furthermore, we established direct interaction between PfHop and PfHsp70-1. Our findings suggest that PfHop interacts with PfHsp70-1 via TPR1 and TPR2B domains. The TPR2A subdomain of PfHop appears only to mediate interaction with PfHsp90. Our findings suggest that PfHop is instrumental in bringing PfHsp70-1 and PfHsp90 into a functional complex to facilitate folding of certain parasite proteins. PfHop has been proposed to be fairly divergent from its human counterpart [19]. Consequently, findings from the current study provide a platform for further investigation of the role of PfHop as a possible antimalarial target.

## Results

### Recombinant protein production and purification

**Fig 1:**
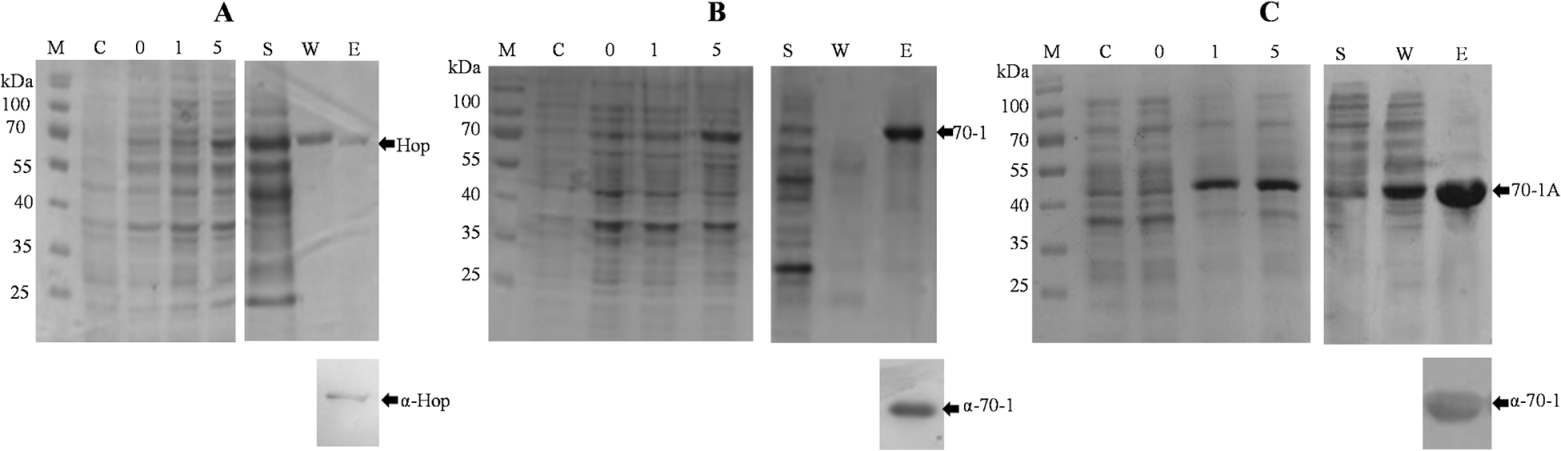
Expression and purification of recombinant PfHop and PfHsp70-1. SDS-PAGE (top panel) representing the purification of PfHop (~ 66 kDa) (panel A); PfHsp70-1 (~ 75 kDa) (panel B) and its ATPase domain (~ 45 kDa) (panel C). Note, the recombinant proteins were attached to an N-terminal histidine tag. The recombinant proteins were over-expressed in *E*. *coli* XL1 Blue cells and purified by nickel-affinity chromatography. Lane M, molecular markers in kDa; Lane C; XL1 Blue cells transformed with pQE30 vector; lane 0, pre-induction sample; lane 1, 1 hour post-IPTG induction sample and lane 5; sample taken 5 hours post-IPTG induction; lane S, supernatant of the whole lysate; lane W, wash; lane E, elution. α-PfHsp70-1 polyclonal antibody (1:2000) was used to confirm the presence of PfHsp70-1 and its ATPase subdomain by Western blotting (lower panels). The arrows indicate bands corresponding to the respective recombinant proteins: PfHop (Hop); PfHsp70-1 (70–1); and the ATPase subdomain of PfHsp70-1 (70-1A).

Recombinant PfHop was expressed in *E*. *coli* XL1 Blue cells as previously described [19] and the his-tagged protein was successfully purified using nickel chromatography (Fig 1A). Similarly, recombinant PfHsp70-1 and its ATPase-subdomain were expressed in *E*. *coli* XL1 Blue cells and both proteins were successfully purified using nickel chromatography (Fig 1B and 1C). Both full-length PfHsp70-1 protein and its ATPase subdomain were recognised by the rabbit-raised anti-PfHsp70-1 polyclonal antibodies as confirmed by Western blot analysis (Fig 1B and 1C).

### PfHop directly interacts with PfHsp70-1

**Fig 2:**
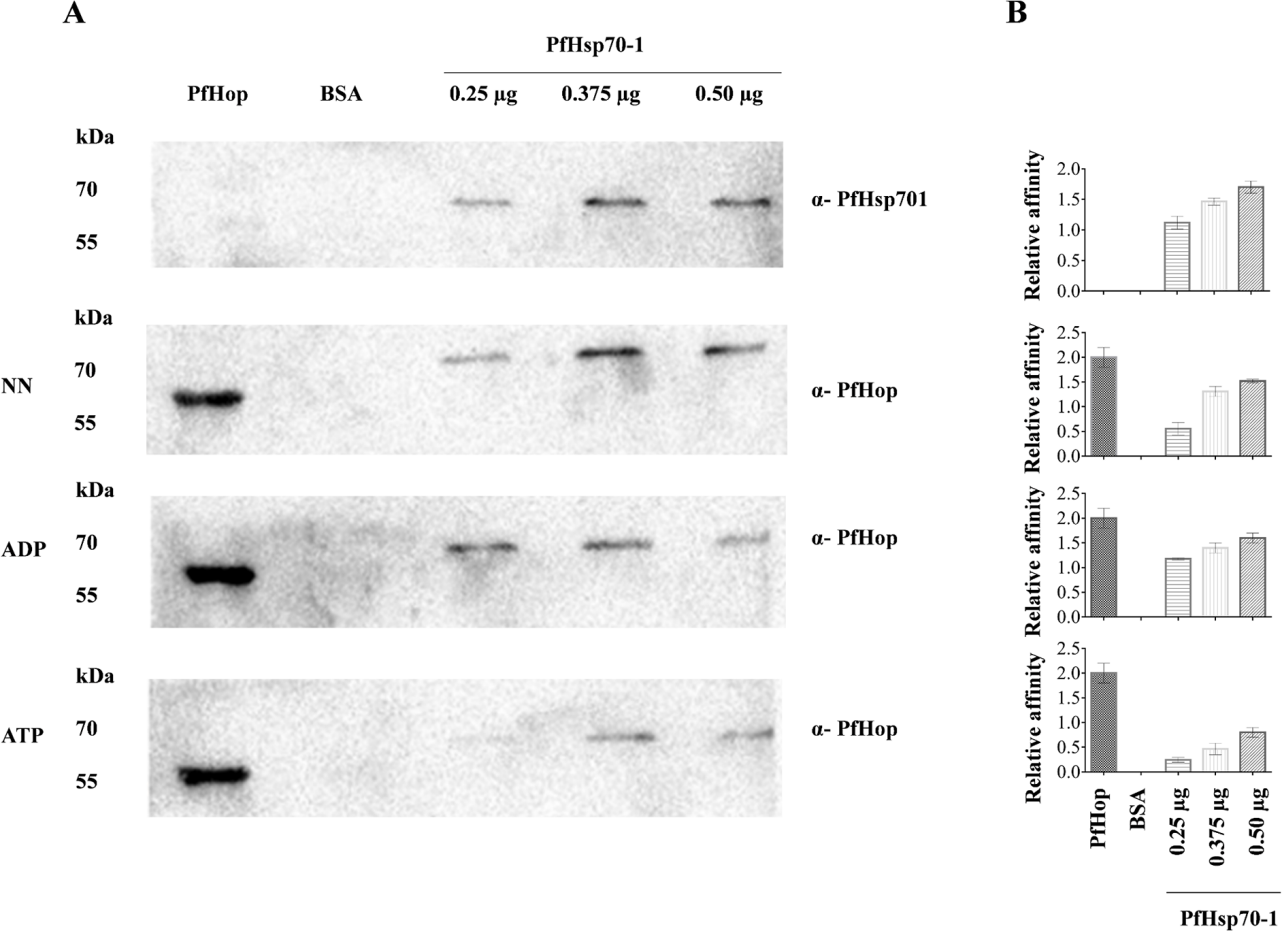
PfHop directly interacts with PfHsp70-1. Interaction between recombinant PfHop and PfHsp70-1 was investigated using far Western analysis. PfHop protein (50 μg/mL); control protein, BSA (50 μg/mL) and PfHsp70-1 protein at various concentrations (25 μg/mL, 37.5 μg/mL, 50 μg/mL) were resolved by SDS-PAGE and transferred to a blot and the blot was probed using α-PfHsp70-1 (A; top panel). A similar blot was then over-layed with PfHop in the absence of nucleotides (A; panel NN); presence of 5 μM ADP (A; panel ADP) and presence of 5 μM ATP (A; panel ATP) and they were probed using α-PfHop. The interaction of PfHop with PfHsp70-1 was validated by densitometric analysis (B). A t-test was used to validate the variation in signal densities representing PfHop bound by PfHsp70-1 in the absence of nucleotide and presence of ATP/ADP (*p<0*.*005*).

Far Western analysis was conducted to investigate the direct interaction of PfHop with PfHsp70-1 (Fig 2). A blot containing PfHsp70-1 (at varying levels) and controls (BSA and PfHop) was probed using α-PfHsp70-1 antibodies which recognised only the PfHsp70-1 protein (Fig 2A; top panel). This confirms that the α-PfHsp70-1 antiserum used was able to distinguish between PfHsp70-1 and PfHop. A similar blot was overlayed with PfHop protein and subsequently probed using α-PfHop antibodies. A protein band was observed in the lane representing PfHop protein (corresponding to protein, ~56.6 kDa in size) (Fig 2A; panel NN). In addition, the α-PfHop antibodies also bound to a species that was around 70 kDa in size corresponding to the lanes into which PfHsp70-1 was under-layed (Fig 2A; panel NN). Thus, the α-PfHop antibodies must have bound to PfHop protein complexed to the PfHsp70-1 protein that was immobilised onto the blot. We have previously demonstrated that the α-PfHop antibodies used in this study are specific for PfHop and do not recognise PfHsp70-1 protein [19]. Therefore the observed association of PfHop with PfHsp70-1 was not due to antibody cross-reactivity. Furthermore, the association was dependent on the amount of PfHsp70-1 protein underlay, suggesting that the interaction was specific as validated by the densitometric analysis (Fig 2A and 2B, panel NN). The assay was repeated in the presence of ADP and ATP and PfHop bound to PfHsp70-1 in the presence of the two nucleotides (Fig 2A; panels ADP/ATP). The observed association of PfHop with PfHsp70-1 in the presence of ATP suggest that the interaction was not based on chaperone-substrate association. However, based on the densitometric analysis, PfHop appeared to interact with PfHsp70-1 less favourably in the presence ATP as compared to its association with the chaperone either in the absence of nucleotide or presence of ADP.

**Fig 3:**
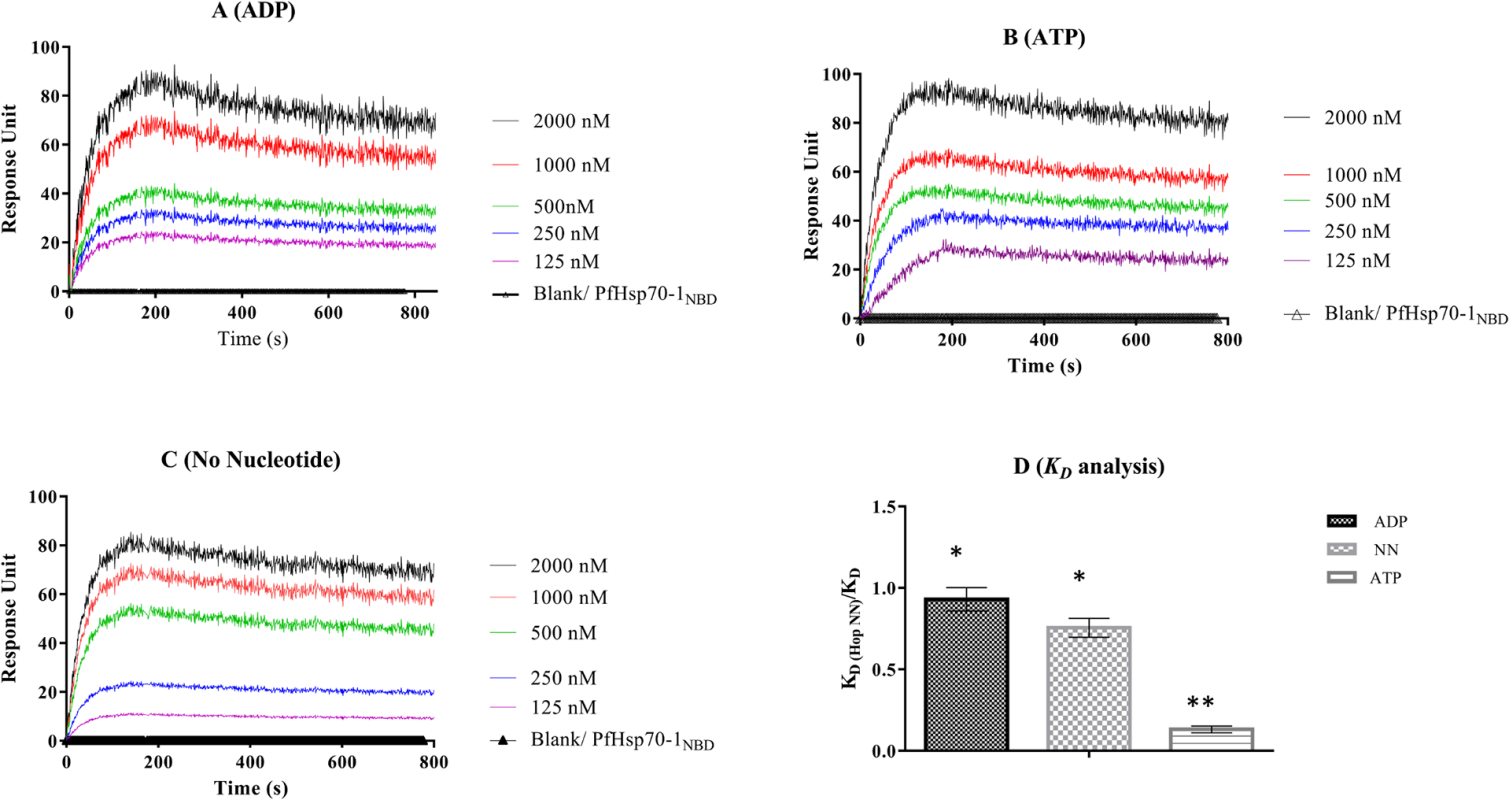
SPR analysis for the interaction of PfHop with PfHsp70-1. To further validate the interaction between PfHop and PfHsp70-1, SPR analysis was conducted with PfHop and PfHsp70-1 alternated as ligand and analyte, respectively. Full length PfHsp70-1 (1 μg/mL) was immobilised on the GLC sensor chip and variable concentrations of PfHop (2 μM, 1 μM, 0.5 μM, 0.25 μM, 0.125 μM) were passed over the immobilised chaperone. The assay was repeated using the ATPase subdomain of PfHsp70-1 (PfHsp70-1_NBD_) as a negative control. The sensograms represent data from at least three independent assays conducted in the presence of 5 mM ATP (panel A), 5mM ADP (panel B) and absence of nucleotides (panel C). The rate constants *k*_*a*_, *k*_*d*_ and *K*_*D*_ values of PfHop towards PfHsp70-1, and PfHop self-association were determined (S2 Table). The relative affinities of PfHop towards PfHsp70-1 and PfHop self-association were determined and presented as bar graphs (panel D). The relative affinities were normalized to PfHop interaction with PfHsp70-1in the absence of nucleotides (NN). Error bars obtained from the t-tests are indicated. We observed no significant difference in the association of PfHop with PfHsp70-1 in the presence of ADP and absence of nucleotide (*), (*p<0*.*005*). However, affinity for the interaction between PfHop and PfHsp70-1 in the presence of ATP was significantly lower than observed in the absence of nucleotide/presence of ADP (**), (*p<0*.*005*).

SPR analysis was further conducted to validate the interaction of recombinant PfHop and PfHsp70-1 proteins. The ATPase subdomain of PfHsp70-1, which lacks the C-terminal EEVD motif crucial for its interaction with Hop [15],) was used as a negative control. Both recombinant forms of PfHsp70-1 (full length) and its ATPase domain were immobilized onto the ProteOn GLC sensor chip. PfHop at varying concentrations was then passed over the immobilized PfHsp70-1. The assay was repeated with analyte and ligand swapped. Based on the SPR analysis, PfHop bound to full length PfHsp70-1 in a concentration dependent fashion (Fig 3). As expected, there was no evidence of interaction between PfHop and PfHsp70-1_NBD_, suggesting that the C-terminus of PfHsp70-1 is crucial for its interaction with PfHop. Since Hsp70 binds to misfolded proteins, we wanted to ascertain that the interaction between PfHsp70-1 and PfHop was based on a chaperone-cochaperone interaction as opposed to a chaperone (PfHsp70-1)-substrate (PfHop) association. Hsp70 releases its substrates when it is bound to ATP and binds its substrate with higher affinity in the ADP-bound state [20]. For this reason, we repeated the SPR assay in the presence of 5 mM ADP or ATP (Fig 3A and 3B). Overall, PfHop protein interacted with PfHsp70-1 in nanomolar range, suggesting a fairly high level affinity (S2 Table). In general, the relative affinities for the interaction of PfHop with PfHsp70-1 in descending order were as follows: presence of ADP>absence of nucleotide>presence of ATP (Fig 3D; S2 Table). The SPR based data thus mirrors the findings obtained by far Western analysis (Fig 2).

**Fig 4:**
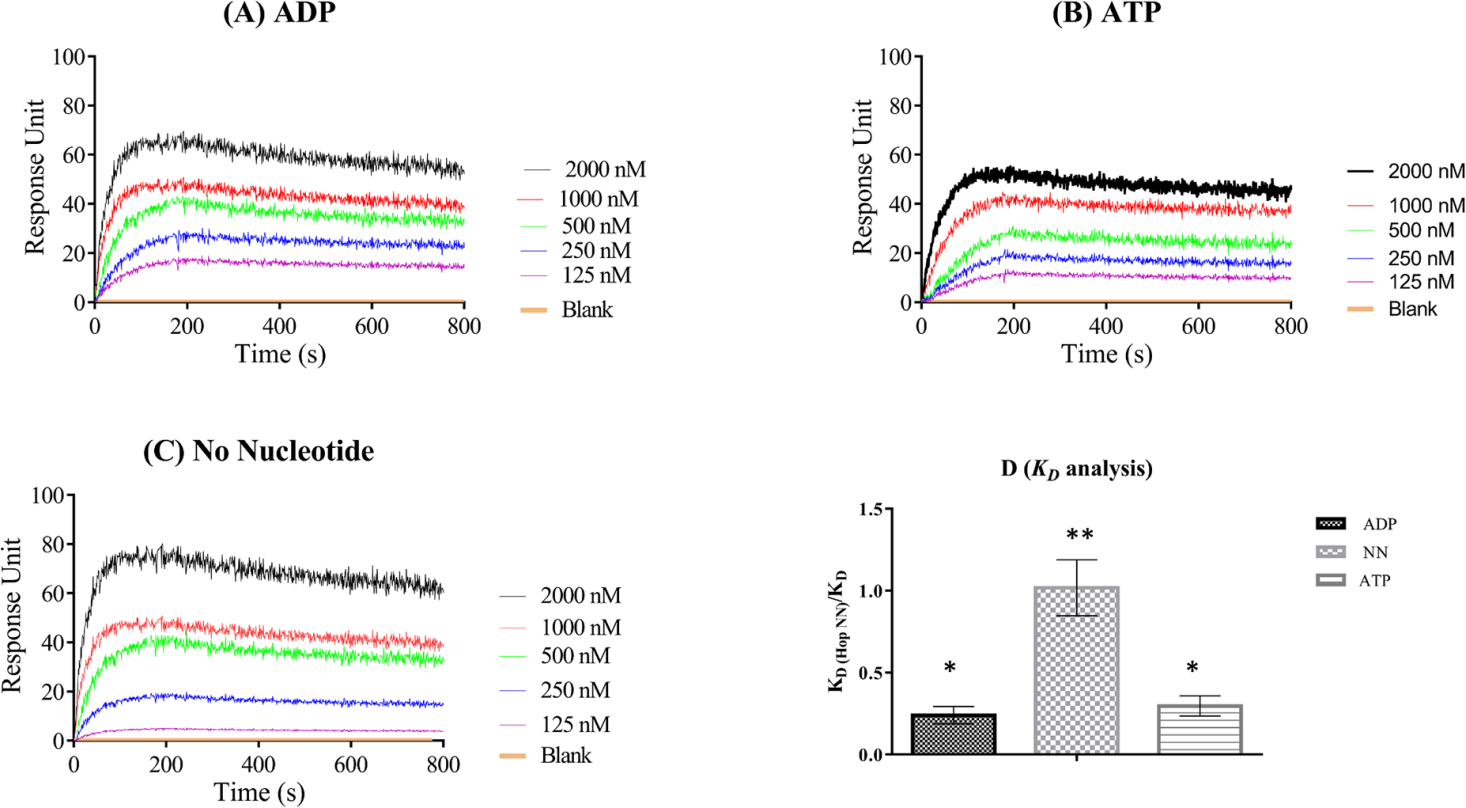
SPR analysis for the self-association of PfHop recombinant protein. Analysis for self-association of PfHop was conducted using SPR analysis with PfHop as both ligand (1 μg/mL) and analyte. Variable amounts of analyte (2 μM, 1 μM, 0.5 μM, 0.25 μM, 0.125 μM) were passed onto the ligand immobilized chip. The PfHsp70-1 (PfHsp70-1_NBD_) was immobilised on the GLC chip as negative control. The sensograms represent data from at least three independent assays conducted in the presence of 5 mM ATP (panel A), 5mM ADP (panel B) and absence of nucleotides (panel C). The rate constants *ka*, *kd* and *K*_*D*_ values of PfHop self-association were determined (S2 Table). The relative affinities of PfHop self-association were determined and presented as bar graphs (panel D). The relative affinities were normalized to PfHop self-association in the absence of nucleotides (NN). Error bars obtained from the t-tests are indicated. We observed no significant difference in the self-association of PfHop in the presence of ADP and presence of ATP (*), (*p<0*.*005*). However, affinity for the self-association of PfHop in the absence of nucleotides was significantly higher than obtained in the presence of nucleotide (**), (*p<0*.*005*).

We further immobilized PfHop onto the chip and investigated its ability to self-associate. As a negative control, we also immobilized PfHsp70-1_NBD_. The SPR sensograms suggested that PfHop associated with itself in a concentration dependent manner (Fig 4). In addition, the association was stable in the absence of nucleotide and also in the presence of either ATP or ADP (Fig 4A–4C). In general, the protein self-associates with affinities in nanomolar range, comparable to the affinity for the previously reported dimerization of human Hsp70 (S2 Table; [21]). However, overall, the association exhibits higher affinity in the absence of nucleotide in comparison to the presence of either ATP/ADP (Fig 4).

### Binding preferences of C-terminal fragments of PfHsp70-1 and PfHsp90 to PfHop TPR subdomains

**Fig 5:**
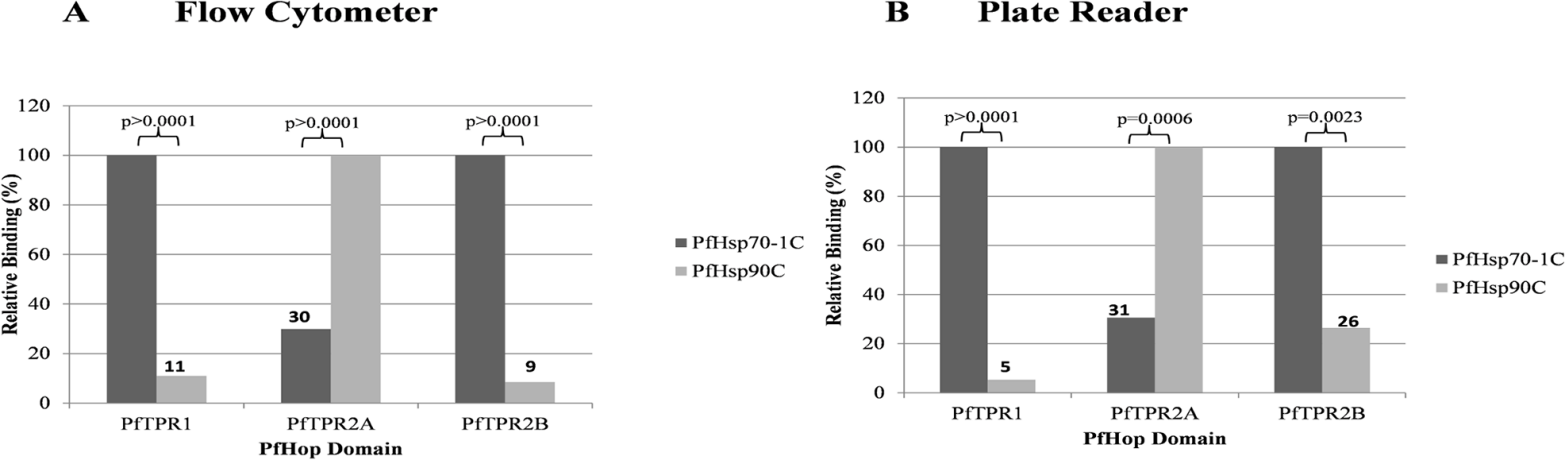
Relative binding preferences of PfHsp70-1 and PfHsp90 C-terminal fragments to TPR domains of PfHop. Fragments encoding the C-terminal fragments of PfHsp90 and PfHsp70-1 were cloned into the pET11a-link-NGFP vector [22]. Fragments encoding TPR1, TPR2A and TPR2B derived from *PfHOP* were cloned into the pMRBAD-link-CGFP vector. The constructs were co-transformed into OneShot BL21 StarDE3, and IPTG was used to induce protein production. The fluorescence signals were measured using either flow cytometry or a fluorescent plate reader. The results (as noted in materials and methods), are expressed as relative, not absolute binding efficiency to each TPR domain. N = 4.

To investigate the relative binding preferences of PfHsp70-1 and PfHsp90 for TPR subdomains of PfHop, we used a split GFP assay. Expression vectors encoding individual PfHop TPR domains (TPR1, TPR2A, TPR2B) fused to C-GFP, were co-expressed in *E*. *coli* with expression vectors encoding the C-terminal domains of either PfHsp70-1 or PfHsp90, fused to N-GFP. Following induction of protein expression and the required incubation at 30°C to allow maturation of the complemented chromophore, we measured relative fluorescence using either flow cytometry or a fluorescent plate reader. The combined results of multiple experimental replicates are presented in (Fig 5). Our data suggest that, similar to the pairing of human TPR domains [22], PfTPR1 preferentially binds Hsp70, whereas PfTPR2A preferentially binds Hsp90. Interestingly, we also observed a differential relative binding affinity to PfTPR2B, with PfHsp70-1 having a much higher binding affinity (Fig 5). This is in agreement with a recent study suggesting that the TPR2B subdomain whose function had remained enigmatic [23] is now known to be the primary binding site of Hsp70 [18]. According to the study by Rӧhl et al, Hsp70 primarily binds to TPR2B [18]. As predicted by the literature, constructs containing full-length PfHop/PfHsp70-1 or PfHsp90 failed to interact, likely due to steric hindrance (data not shown) [22].

### PfHop is stress-inducible protein

**Fig 6:**
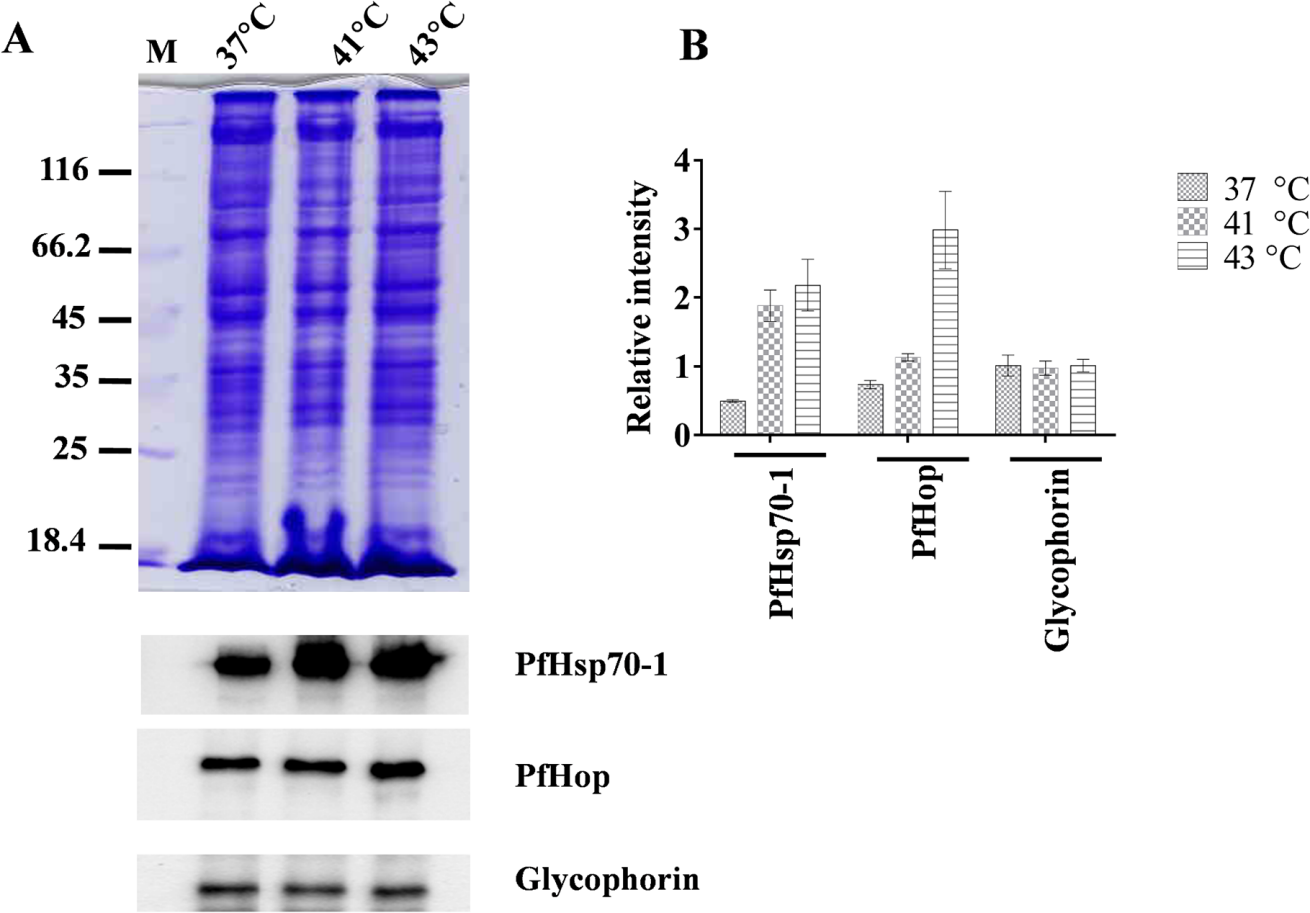
PfHop is heat-inducible. The expression of PfHop and PfHsp70-1 was investigated on total cell lysate isolated from parasites cultured *in vitro* at 37°C, 41°C or 43°C. Samples were taken from cultures that were left to grow at the respective temperature for either 1 hour. The cell lysate was resolved by SDS-PAGE and Western blot analyses using antibodies recognizing PfHop, PfHsp70-1 and glycophorin (loading control), respectively.

PfHsp70-1 and PfHsp90 are stress-inducible molecular chaperones [5,8]. Since PfHop is proposed to coordinate the functional partnership of these two prominent molecular chaperones, we investigated its expression in parasites cultured *in vitro*. The expression of PfHop by parasites at the trophozoite stage was examined under normal conditions (37°C) and heat shock conditions (41°C and 43°C), respectively, for 1 hour. As expected, PfHsp70-1 expression was induced in parasites that were exposed to heat stress conditions (41°C and 43°C) (Fig 6). Similarly, parasites that were cultured for 1 hour at 41°C and 43°C over-produced both PfHop and PfHsp70-1 compared to parasites that were exposed to 37°C for 1 hour (Fig 5). This indicates that PfHop, like PfHsp70-1 [5], is induced by heat stress.

### PfHop occurs in a complex with PfHsp70-1

**Fig 7:**
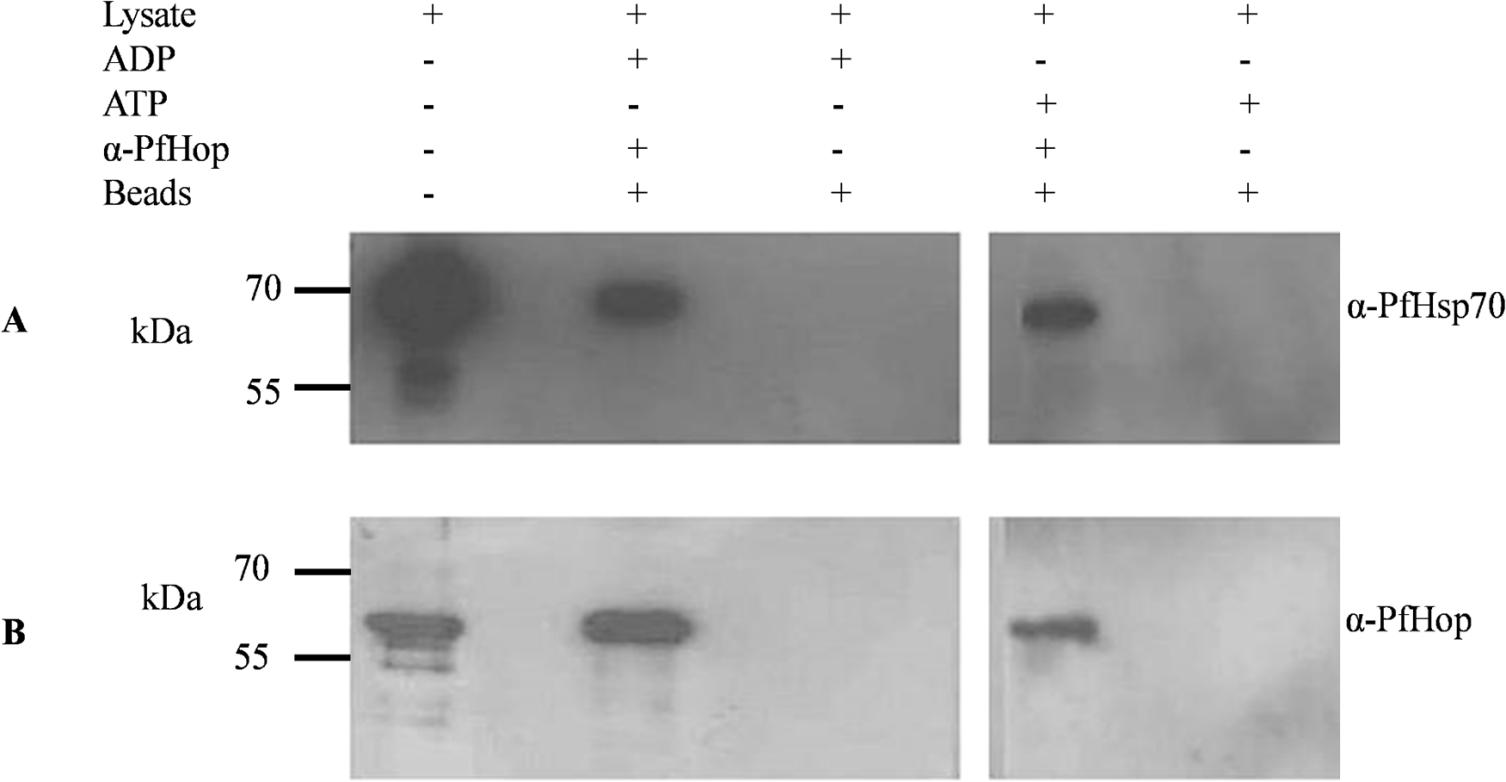
PfHop co-immunoprecipitates with PfHsp70-1 in the presence of either ATP or ADP. α-PfHop antibodies were used in the co-immunoprecipitation (Co-IP) step and α -PfHsp70-1 and α -PfHop antibodies were used for Western analysis (panel A and B), respectively. Lane 1, whole parasite lysate prior to co-IP; lane 2, Co-IP conducted in presence of 5 mM ADP/ATP.

PfHop and PfHsp90 are known to associate [19,24]. However, An *et al* [24] could not establish an association between Hop and Hsp70 in *P*. *falciparum*. Based on a co-immunoprecipitation assay, we previously noted that the interaction of PfHop with PfHsp70-1 in the presence or absence of ATP [19]. In the current study, we repeated the co-immunoprecipitation assay on the parasite lysate using α-PfHop antibodies either in presence of ATP or ADP. The association of PfHop and PfHsp70-1 occurred in the presence of both ADP and ATP (Fig 7). Therefore, the co-immunoprecipitation data further validated findings obtained based on the far Western and SPR analyses (Figs 2 and 3).

## Discussion

Although the association between PfHop and PfHsp90 is fairly well established [19,24], the direct interaction between PfHop and PfHsp70-1 has until now not been confirmed. Findings from the current study suggest that PfHop and PfHsp70-1 interact in a nucleotide dependent fashion. The findings further confirmed that this interaction occurs via the C-terminal peptide binding domain of PfHsp70-1 as PfHsp70-1 lacking the C-terminus was unable to bind PfHop based on SPR analyses (Fig 3). It is known that Hop primarily binds to the EEVD motif located at the C-terminus of Hsp70 [15]. Therefore, the observed interaction between PfHop and PfHsp70-1 was specific and not a general protein-protein association. Although we previously demonstrated the association of PfHop with PfHsp70-1 and PfHsp90 by co-immunoprecipitation based assays, the current findings provide the first direct evidence for the association of PfHop with full length PfHsp70-1. In addition, we also observed interaction between C-terminal fragments of PfHsp70-1/PfHsp90 with TPR subdomains of PfHop.

Using SPR analysis and far western blotting, we observed that the association of PfHop with PfHsp70-1 is favored by ADP compared to ATP (Figs 2, 3 and 4; S2 Table). This is in line with observation made on the interaction of Hop and Hsp70 from other species [25]. It is known that the C-terminal domain of Hsp70 in which the EEVD motif is located maintains a similar conformation in the presence of ADP and in a nucleotide-free form of the chaperone [26]. Therefore, it is not surprising that nucleotide-free and ADP-bound forms of PfHsp70-1 interact with PfHop better than the ATP-bound form of the chaperone (Fig 2; S2 Table). It has further been reported that the presence of increasing levels of Hsp90 reduces the interaction between Hop and Hsp70 [17]. In addition, it has been suggested that Hsp70 primarily binds to the TPR2B subdomain of Hop in the absence of Hsp90 [18]. Based on the latter hypothesis, upon Hsp90 binding, Hsp70 switches to the TPR1 module of Hop. It has also been suggested that increasing levels of Hsp90 competitively lowers the amount of Hsp70 bound to Hop [17]. This is difficult to conceive since Hop binds to Hsp70 and Hsp90 via independent domains. Making the role of TPR domains of Hop more intriguing is the observation that a Hop protein from *Caenorhabditis elegans* (CeHop) which lacks a TPR1 domain is capable of binding to both Hsp70 and Hsp90. [27]. In light of the contrasting views with respect to the role of TPR domains of Hop from various species, we were prompted to investigate the role of the TPR domains of PfHop in mediating its interaction with PfHsp70-1 and PfHsp90. Using a split GFP assay [22], we established that C-terminal EEVD motif of PfHsp70-1 interacts with the TPR1 and TP2B subdomains of PfHop (Fig 5). On the other hand, the TPR2A subdomain of PfHop bound to the C-terminal fragment of PfHsp90 (Fig 5). This is in agreement with findings from a previous study which proposed that Hsp70 is capable of binding to Hop via both the TPR1 and TPR2B subdomains [16]. Consequently, we hypothesize that PfHsp70-1 binds to PfHop through the TPR1-DP1 in the absence of PfHsp90 as has been previously proposed for its Sti1 homologue [27]. Therefore, it is possible that binding of PfHsp90 would promote PfHsp70-1 to switch to the TP2B-DP2 module of PfHop to facilitate substrate transfer between the two chaperones.

A Hop homologue has been reported to form a stable monomer, and exhibited a less stable dimer form [28]. In the current study, we established that PfHop is capable of self-association at nanomolar levels, exhibiting affinity that is comparable to the reported affinities for the dimerization of human Hsp70 (S2 Table; [21]). We noted no significant difference in the self-association of PfHop in the presence of ADP compared to the presence of ATP. However, affinity for the self-association of PfHop in the absence of nucleotides was significantly higher than observed in the presence of nucleotides. In support of this, it has been recently reported that Hop binds ATP, and that the protein exhibits ATPase activity [29]. Therefore nucleotides may regulate not only the self-association of PfHop but may have broader effects on the function of this protein.

The development of malaria parasites at the blood stage is characterised by several physiologically distinct growth stages. Furthermore, the development of malaria is associated with periodic fevers. In response to variation in physiological conditions, malaria parasites are capable of modulating the expression of some of their heat shock proteins in order to meet the prevailing protein folding demands. Furthermore, it has been proposed that the expression of some heat shock proteins correlate with the prognosis of clinical malaria [12]. Based on our findings PfHop expression is stress-induced. Similarly, the expression of PfHsp70-1 and PfHsp90 is induced by heat stress [5,30]. The stress-inducible expression of PfHop points to an important role in coordinating the functional partnership between PfHsp70-1 and PfHsp90 under conditions that are unfavourable to proteostasis such as periodic fever conditions that characterise clinical malaria.

PfHsp70-1 and PfHsp90 are both potential antimalarial drug targets [8,10,31]. A study based on a mouse malaria model, showed that an inhibitor of PfHsp90 complemented the antimalarial action of chloroquine [11], suggesting that targeting Hsp90 function could reverse drug resistance by the parasite. It has previously been reported that both chaperones associate with a malarial protein, ferriprotoporphyrin IX [32], which is implicated in the development of chloroquine resistance [33]. This further suggests a possible role of these two chaperones in the development of drug resistance. Therefore, characterisation of the PfHsp70-1-PfHop-PfHsp90 pathway may provide an additional step towards the design of antimalarial compounds targeting the function of PfHop. PfHop is predicted to be structurally divergent from the human homologue, and therefore may be selectively inhibited with minimum effects on the function of human Hop [19]. There is increasing evidence that heat shock proteins from the malaria parasite may be inhibited by some small molecules, which target them selectively with minimum effects on human heat shock proteins [31,34]. Part of our broad spectrum of ideas is to establish the heat shock protein network that is crucial for the survival of malaria parasites. To this end, we recently characterised an Hsp110 homologue from *P*. *falciparum* (PfHsp70-z) whose possible function is to facilitate nucleotide exchange function of PfHsp70-1 [35]. Therefore, findings from this study contribute towards mapping out the heat shock protein network of malaria parasites towards establishing possible antimalarial targets. To this end, we intend to optimise the GFP-split based assay, we developed in the current study towards screening antimalarial compounds that target the PfHop coordinated protein folding partnership between PfHsp70-1 and PfHsp90.

## Materials and Methods

### Antibodies

To confirm the expression of PfHop we used previously described antibodies which were generated by immunising rabbits with a synthetic peptide, TGEGNDAEERQRQQR, corresponding to amino acids 195–206 of the PfHop sequence [19]. The expression of PfHsp70-1 was confirmed by Western blotting using rabbit raised anti-PfHsp70-1 antibodies that were described in previous studies [5,19].

### Cloning of the ATPase subdomain of PfHsp70-1

The interaction between Hop and Hsp70 occurs via the C-terminally located EEVD motif of the latter [15]. As a control, a subdomain of PfHsp70-1, excluding the C-terminal peptide binding domain of the protein was cloned and expressed. A construct expressing PfHsp70-1 (pQE30/PfHsp70-1) which was previously used to express the protein in *E*. *coli* [20] was used as a template and primers were designed to place a stop codon immediately downstream of the bases encoding for the residue S398 which marks the end of the ATPase domain of PfHsp70-1. Site directed mutagenesis was used to introduce the stop codon. The following primers were used: forward primer: 5′-GGTGACCAATCATA**A** **CTAGT**CCAAGATTTATTATTATTAG-3′; and the reverse primer: 5′- CTAATAATAATAAATCTTGG**ACTAG** **T**TATGATTGGTCACC-3′ (stop codon underlined). A *Spe*I restriction site (in bold) was added downstream of the stop codon. The insertion of the stop codon was confirmed by restricting the construct using *Spe*I and the DNA was resolved by agarose gel electrophoresis. The integrity of the construct was further confirmed through DNA sequencing.

### Expression and purification of recombinant proteins

A construct expressing PfHop (pQE30/PfHop) which was previously described [19] was used to express PfHop protein in *E*. *coli* XL1 Blue cells. Similarly, *E*. *coli* XL1 Blue cells were also used to express PfHsp70-1 and its ATPase derivative following a previously described protocol [20]. The proteins were expressed with N-terminal polyhistidine tags in order to facilitate their purification using nickel affinity chromatography. The expression and purification of the recombinant proteins was verified by electrophoresis using 12% sodium duodecyl sulphate polyacrylamide gel electrophoresis (SDS-PAGE), followed by Western blot analysis using anti-PfHop.

### Investigation of the interaction between PfHop and PfHsp70-1 by far Western analysis

A far Western analysis was conducted as previously described [36] with slight modifications. Briefly, various amounts (25 μg/mL, 37.5 μg/mL and 50 μg/mL) of recombinant PfHsp70-1 protein and recombinant PfHop protein (50 μg) as well as 50 μg of BSA (negative control) were resolved using 12% SDS PAGE. The proteins were subsequently transferred onto Hybond ECL nitrocellulose membrane (GE Healthcare). The proteins on the membrane were denatured and renatured by using various levels of urea (8 M– 0 M). The membranes were blocked using 5% fat free milk in 1X Tris-buffered saline (TBS; 50 mM Tris-HCl, pH 7.5, 150 mM NaCl and 0.1% Tween 20) for 1 hour at room temperature. The blot was then incubated with the ligand protein (50 mg/ml of PfHop) in protein-binding buffer (100 mM NaCl, 20 mM Tris [pH 7.6], 0.5 mM EDTA, 10% glycerol, 0.1% Tween-20, 2% skim milk powder and 1 mM DTT) [36] overnight at 4°C. The procedure was repeated by exposing the blot to no nucleotides and in other instances 5 μM ATP or ADP added onto two respective blots. A control blot was incubated in binding buffer lacking ligand. After washing three times in TBS-Tween buffer (0.1% Tween-20 in 1X Tris-buffered saline pH 7.5), the blots were incubated in 3% milk (w/v) in the presence of anti-PfHop antibodies (1: 2000) for 2 hours at 4°C. One replicate blot was incubated with α-PfHsp70-1 antibody (1:2000). After subsequent washing steps, the membranes were incubated with the horseradish peroxidase conjugated secondary antibody for 2 hours at 4°C. Unbound secondary antibodies were removed by washing the membrane twice using TBS-Tween buffer. The membrane bound secondary antibody was detected using ECL chemiluminescence reagent (Thermo Scientific) and Images were acquired using ChemiDoc Imaging system (Bio-Rad, USA) and densitometric analysis for the respective protein bands were conducted using the Image Lab version 5.1 built 8 (Bio-Rad, USA).

### Analysis of the PfHop-PfHsp70-1 interaction and PfHop self-association using surface plasmon resonance

Surface plasmon resonance (SPR) analysis was performed using a ProteOn XPR36 Interaction Array System with a GLC sensor chip (Biorad, USA). The assay was conducted at room temperature (25°C). The reaction mix was suspended in filter sterilised and degassed PBS-Tween buffer (4.3 mM Na_2_HPO_4_, 1.4 mM KH_2_PO_4_, 137 mM NaCl, 27 mM KCl, 0.005% Tween-20 and 20 mM EDTA; pH 7.4). PfHsp70-1 and PfHop were covalently attached to the modified alginate polymer layer on the GLC sensor chip via amine coupling following a protocol that was provided by the manufacturer (ProteOn XPR36, Bio-rad, USA). Recombinant PfHop (0.5 μg/mL) and PfHsp70-1/ PfHsp70-1_NBD_ (1 μg/mL) were immobilised onto the chip. At these concentrations of the proteins, the following response units (RU) were obtained: 187 RU for PfHop, 196 RU for PfHsp70-1 and 198 RU for PfHsp70-1_NBD._ As analytes, aliquots of PfHop, PfHsp70-1 and PfHsp70-1_NBD_ were prepared at final concentration of 2000-, 1000-, 500-, 250-, and 125 nM were injected at 50 μl/min into each horizontal channel. Association was allowed for 2 min, and dissociation was monitored for 10 min. Association (*k*_*a*_), dissociation (*k*_*d*_), and equilibrium (*K*_*D*_) rate constant data were processed and analysed using ProteOn Manager Software version 3.1.0.6. by concatenating the responses of all five analyte concentrations. PfHsp70-1 ATPase domain was used as a negative control. Self-association of PfHop was investigated following the same protocol using recombinant PfHop protein as ligand and analyte.

### Generation of split-GFP constructs

Fragments encoding the C-terminal (24 residues) subunits of PfHsp90 and PfHsp70-1 were cloned into the pET11a-link-NGFP vector [22]. Fragments encoding TPR1, TPR2A and TPR2B derived from PfHOP were cloned into the pMRBAD-link-CGFP vector [22]. All vector constructs were verified by automated sequencing (GATC, Konstanz, Germany) and restriction digest. Primers used for PCR amplification of all fragments from genomic DNA (clone 3D7) are listed in (S1 Table).

### Measurement of bimolecular fluorescence complementation (BiFC)

Final constructs were co-transformed into OneShot BL21 StarDE3 (Life technologies). Protein expression was induced as previously described, using IPTG and chloramphenicol [22]. Fluorescence was measured either by flow cytometry or using a fluorescent plate reader using appropriate filters and excitation wavelengths. The reported values represent means obtained from three independent experiments carried out on separate days that were expressed after subtraction of background fluorescence (cells expressing only N- and C- terminal GFP fragments, without potential interaction partners). Binding of C-terminal fragments of PfHsp70 and PfHsp90 to TPR motifs of PfHop was reported relative to the highest measured fluorescence and is thus referred to as "relative binding". P-values to validate the relative affinities of PfHop TRP domains for C-terminal fragments of PfHsp70-1 and PfHsp90 were calculated using the student’s t-test.

### Assessment of the expression of PfHop in response to heat stress

*P*. *falciparum* 3D7 cells were cultured in human O^+^ erythrocytes according to standard protocol [37]. The blood was provided by the University of Marburg Blood Bank. Red blood cells infected by *P*. *falciparum* 3D7 parasites were suspended in RPMI 1640 containing L-Glutamine, HEPES, 0.2 mM hypoxanthine, 0.1 mg/ml neomycin, 10% (v/v) human plasma, and 2.4% (v/v) human erythrocytes. The culture was exposed to an atmospheric composition of 3% (v/v) CO_2_, 4% (v/v) O_2_ and 93% (v/v) N_2._ The temperature was maintained at 37°C. Synchronised parasites were harvested using gelafundin floatation at the trophozoite stage which was then cultured to ring stage. To investigate the heat-inducible expression of PfHop, red blood cells infected by parasites at the trophozoite stage (5% parasitaemia) were cultured in duplicates. One batch of the culture was incubated at 37°C, while two others were incubated at 41°C and 43°C, respectively. The cultures were incubated for 1 hour. Cells were harvested at the trophozoite stage and were washed in PBS (pH 7.4). The erythrocyte membrane was lysed in PBS containing saponin 0.1% (w/v). Lysis was conducted on ice for 10 minutes with gentle mixing after every 2 minutes. Parasites were collected by centrifugation at 2,500 g for 4 minutes followed by three washes using PBS (pH 7.4). Thereafter, the parasite lysate was subjected to SDS-PAGE and Western analysis. Levels of PfHop, PfHsp70-1 and glycophorin were determined by Western blotting using antibodies that recognized the respective proteins. The images that were generated from the Western blots analysis were acquired using the Bio-Rad’s VersaDoc Model 4000 imaging system.

### Co-immunoprecipitation of PfHop with PfHsp70-1

Approximately 1x10^9^ parasites harvested at the trophozoite stage were resuspended in 0.1% w/v PBS containing saponin. The suspension was subjected to gentle agitation on ice for 10 minutes. After extensive washing with PBS pH 7.4, the parasites were resuspended in 500 μl immunoprecipitation lysis buffer (0.025 M Tris, 0.15 M NaCl, 0.001 M EDTA, 1% NP-40, 5% glycerol; pH 7.4, containing 1 mM PMSF). Parasite lysate containing approximately 300 μg of total protein were suspended in an aminolink plus coupling resin to which anti-PfHop antibodies had been attached (Pierce, Thermo scientific). Binding was allowed to occur for 2 hours at 4°C with gentle agitation. To investigate the effect of nucleotide on PfHop binding to PfHsp70-1, the suspension was split into two. One aliquote was adjusted to 5 mM ATP and the other to 5 mM ADP. Following subsequent washing steps, the immunoprecipitate was eluted in 150 μl of elution buffer (50 mM glycine, pH 2.8) and thereafter concentrated using 10% (v/v) trichloroacetic acid. The immunoprecipitate was analyzed by Western blot to probe for PfHsp70-1 and PfHop using anti-PfHsp70-1 and anti-PfHop antibodies, respectively.

## Supporting Information

S1 Fig Subdomains of Hsp70, Hsp90 and Hop. Schematic illustrating various domains of Hsp70, Hsp90 and Hop, respectively. “N” represents the N-terminus and “C” represents the C-terminus. Both Hsp70 and Hsp90 possess the ATPase domain (nucleotide binding domain), a linker, a substrate binding domain (SBD) and a C-terminal EEVD motif. Hsp90 possesses an additional middle domain and a dimerization domain. Hop is comprised of three tetracopeptide domains: TPR1, TPR2A, TPR2B and two dipeptide repeats (DP): DP1 and DP2.
(TIF)

S1 Table Primer sequences. (DOCX)

S2 Table Data for the kinetics of PfHop-PfHsp70-1 interaction and PfHop self-association. (DOCX)
