# Supplementary figures and images for: Plasmodium falciparum Hop (PfHop) Interacts with the Hsp70 Chaperone in a Nucleotide-Dependent Fashion and Exhibits Ligand Selectivity
Source: PLoS One. 2015 Aug 12;10(8):e0135326. doi: 10.1371/journal.pone.0135326 (PMC4534038; doi:10.1371/journal.pone.0135326)

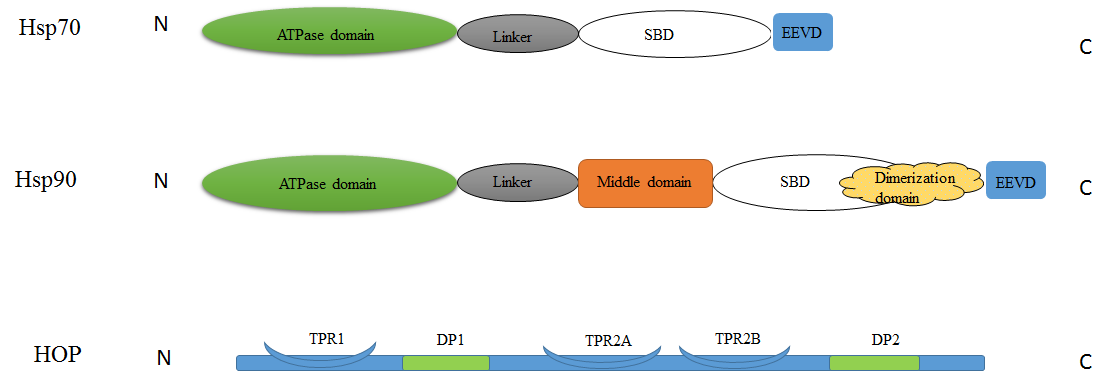

Supplement: S1 Fig — Schematic illustrating various domains of Hsp70, Hsp90 and Hop, respectively. “N” represents the N-terminus and “C” represents the C-terminus. Both Hsp70 and Hsp90 possess the ATPase domain (nucleotide binding domain), a linker, a substrate binding domain (SBD) and a C-terminal EEVD motif. Hsp90 possesses an additional middle domain and a dimerization domain. Hop is comprised of three tetracopeptide domains: TPR1, TPR2A, TPR2B and two dipeptide repeats (DP): DP1 and DP2. (TIF) [file pone.0135326.s001.tif]
