# Supplementary material for: Plasmodium falciparum Hop (PfHop) Interacts with the Hsp70 Chaperone in a Nucleotide-Dependent Fashion and Exhibits Ligand Selectivity
Source: PLoS One. 2015 Aug 12;10(8):e0135326. doi: 10.1371/journal.pone.0135326 (PMC4534038; doi:10.1371/journal.pone.0135326)
Supplement: S1 Table — (DOCX) [file pone.0135326.s002.docx]

**S1 Table. Primer sequences**

| **Designation** | **5‘🡪3‘** |
| --- | --- |
| **TPR1_NcoI_F** | AATAATCCATGGCTGAAGCTCAGAGATTAAAAGAATTAG |
| **TPR1_AatI_R** | AATAAGACGTCCCTGCATTTTCTAACATATTTTCATTCC |
| **TPR2A_NcoI_F** | AATAATCCATGGCTCAAGGTGATGAACATAAATTAAAAGG |
| **TPR2A_AatI_R** | AATAAGACGTCCCTTTTTCTTTTCTTCTTTCTAATTCTTTTAAAG |
| **TPR2B_NcoI_F** | AATAATCCATGGCTGATAAAGCTGAGGAACATAAAAATAAAGG |
| **TPR2B_AatI_R** | AATAAGACGTCCCATCTATTTTGAATGCACATCTTTGATATC |
| **Hsp70-1_C_X_F** | AATAATCTCGAGAGATGCTGCTGGTGCAGCC |
| **Hsp70-1_Cterm_Ba** | AATAATGGATCCTTAATCAACTTCTTCAACTGTTG |
| **Hsp90_C_X_F** | AATAATCTCGAGAGAAGAAAACAATGATATCGATTTACC |
| **Hsp90_Cterm_BamI** | AATAATGGATCCTTAGTCAACTTCTTCCATTTTAGAATCG |

**S1 Table legends:** Sequences for the primers that were used to clone GFP-tagged C-terminal fragements of PfHsp70-1/ PfHsp90 and TPR domains of PfHop
