# Supplementary material for: Plasmodium falciparum Hop (PfHop) Interacts with the Hsp70 Chaperone in a Nucleotide-Dependent Fashion and Exhibits Ligand Selectivity
Source: PLoS One. 2015 Aug 12;10(8):e0135326. doi: 10.1371/journal.pone.0135326 (PMC4534038; doi:10.1371/journal.pone.0135326)
Supplement: S2 Table — (DOCX) [file pone.0135326.s003.docx]

**S2 Table. Data for the kinetics of PfHop -PfHsp70-1 interaction and PfHop self-association**

| Analyte | Ligand | Nucleotide | *ka* (1/Ms) [E+03] | *kd* (1/s)  [E-04] | *K_D_* (nM) | *Χ^2^* | Reference |
| --- | --- | --- | --- | --- | --- | --- | --- |
| PfHop | PfHsp70-1 | ADP | 3.50 (+/-0.1) | 3. 72 (+/- 0.05) | 1.72 (+/-0.3) | 2.3 | This study |
| PfHop | PfHsp70-1 | - | 8.70 (+/-0.06) | 1.66 (+/- 0.02) | 1.91 (+/- 0.1) | 2.17 | This study |
| PfHop | PfHsp70-1 | ATP | 6.95 (+/- 0.09) | 7.83 (+/-0.1) | 11.30 (+/-0.5) | 2.15 | This study |
| PfHop | PfHop | ADP | 59.1 (+/-0.9) | 4.64 (+/-0.02) | 7.85 (+/-0.5) | 2.49 | This study |
| PfHop | PfHop | - | 228 (+/- 2) | 3.59 (+/-0.09) | 1.57 (+/- 0.7) | 0.89 | This study |
| PfHop | PfHop | ATP | 63.5 (+/-0.9) | 3.29 (+/-0.08) | 5.18 (+/-0.1) | 1.84 | This study |
| PfHop | PfHsp70-1_NBD_ | ATP/ADP | ND | ND | ND | - | This study |
| PfHop | PfHsp70-1_NBD_ | - | ND | ND | ND | - | This study |
| hHsp70 | hHsp70 | - | - | - | 4.50 | - | [24] |
| hHsp70 | hHsp40 | - | - | - | 0.50 | - | [24] |

**S2 Table legend**: SPR kinetic data for interaction of PfHop with PfHsp70-1.PfHop and PfHsp70-1 were alternately used as ligand and analyte either in the absence of nucleotide or presence of 5mM ATP/ADP. To investigate PfHop self-association, the protein was mounted on the GLC chip and another aliquote of PfHop was used as analyte. Shown in parenthesis are the standard errors of measurements. ‘ND’ represents not determined, Note, there is no evidence for interaction between PfHop and the PfHsp70-1_NBD_ in the absence or presence of nucleotides. The Χ^2^ demonstrates the goodness of fit for the model on the raw data fitting to models generated during analysis.
